# Preoperative low-dose ketamine has no preemptive analgesic effect in opioid-naïve patients undergoing colon surgery when nitrous oxide is used - a randomized study

**DOI:** 10.12688/f1000research.5258.1

**Published:** 2014-09-23

**Authors:** Beatriz Nistal-Nuño, Enrique Freire-Vila, Francisco Castro-Seoane, Manuel Camba-Rodriguez

**Affiliations:** 1 Department of Anesthesiology, Complexo Hospitalario Universitario A Coruña, A Coruña, Spain; 2 Department of Anesthesiology, Complexo Hospitalario Arquitecto Marcide - Profesor Novoa Santos, Ferrol, Spain

**Keywords:** colon surgery, ketamine, patient-controlled-analgesia, preemptive analgesia

## Abstract

**Background: **The analgesic properties of ketamine are associated with its non-competitive antagonism of the N-methyl-D-aspartate receptor; these receptors exhibit an excitatory function on pain transmission and this binding seems to inhibit or reverse the central sensitization of pain. In the literature, the value of this anesthetic for preemptive analgesia in the control of postoperative pain is uncertain. The objective of this study was to ascertain whether preoperative low-dose ketamine reduces postoperative pain and morphine consumption in adults undergoing colon surgery.

**Methods: **In a double-blind, randomized trial, 48 patients were studied. Patients in the ketamine group received 0.5 mg/kg intravenous ketamine before surgical incision, while the control group received normal saline. The postoperative analgesia was achieved with a continuous infusion of morphine at 0.015 mg∙kgˉ¹∙hˉ¹ with the possibility of 0.02 mg/kg bolus every 10 min. Pain was assessed using the Visual Analog Scale (VAS), morphine consumption, and hemodynamic parameters at 0, 1, 2, 4, 8, 12, 16, and 24 hours postoperatively. We quantified times to rescue analgesic (Paracetamol), adverse effects and patient satisfaction.

**Results:** No significant differences were observed in VAS scores between groups (P>0.05), except at 4 hours postoperatively (P=0.040). There were no differences in cumulative consumption of morphine at any time point (P>0.05). We found no significant differences in incremental postoperative doses of morphine consumption in bolus, except at 12 h (P =0.013) and 24 h (P =0.002). The time to first required rescue analgesia was 70 ± 15.491 min in the ketamine group and 44 ± 19.494 min in the control (P>0.05). There were no differences in hemodynamic parameters or patient satisfaction (P>0.05).

**Conclusions: **Preoperative low-dose-ketamine did not show a preemptive analgesic effect or efficacy as an adjuvant for decreasing opioid requirements for postoperative pain in patients receiving intravenous analgesia with morphine after colon surgery.

## Introduction

In spite of the techniques we have at our disposal and the elementary nature of incisional pain, optimal pain management remains a challenge ^1^. Because the severity of early postoperative pain relates to residual pain after some types of surgery, perioperative pain management can considerably influence the long-term quality of life in patients ^2, 3^.

Woolf, in 1983, first introduced the theory of preemptive analgesia to attenuate postoperative pain ^4^, confirming the presence of a central factor of post-injury pain hypersensitivity in experimental research. After this, experimental studies showed that various anti-nociceptive methods applied before injuries were more effective in reducing post-injury central sensitization in contrast to administration after injury ^5^.

After activation of C-fibers by noxious stimuli, sensory neurons become more sensitive to peripheral inputs, a process called central sensitization ^6, 7^. ‘Wind up’ ^8^, another mechanism activating spinal sensory neurons, is seen after reiterated stimulation of C-fibers. These sensitizations produce c-fos expression in sensory neurons ^9^, and are related to the activation of N-methyl-D-aspartic acid (NMDA) ^7, 9^ and neurokinin receptors ^10, 11^. These genes produce long-lasting changes in the pain-processing system, resulting in hyperexcitation. According to Wall, protection of sensory neurons against central sensitization may provide relief from pain after surgery ^12^. Based on this assumption, preemptive analgesia has been recommended as an effective aid to control postsurgical pain ^4, 13, 14^. NMDA antagonists have been demonstrated to block the induction of central sensitization and revoke the hypersensitivity once it is established ^7, 15^.

Ketamine is an old drug that is increasingly being considered for the treatment of acute and chronic pain. Its pharmacology and mechanism of action as an NMDA receptor antagonist are adequately known, but in clinical practice it presents irregular results ^16^. Since ketamine is an NMDA-receptor antagonist, it is supposed to avoid or revoke central sensitization, and thus to attenuate postoperative pain ^17^.

This antihyperalgesic action can be achieved by smaller doses than those required for anesthesia. Small-dose ketamine has been specified as not more than 1 mg/kg when given as an iv bolus, and not higher than 20 µg∙kg ^-1^∙min ^-1^ when given as a constant infusion ^18, 19^.

Low-doses preemptive ketamine administered iv seem to reduce postoperative pain and/or analgesic consumption ^15, 20, 21^. According to one study ^19^, a single dose of ketamine 1 mg/kg, when administered in conjunction with local anesthetics, opioids or other anesthetics, provides good postoperative pain control ^17^.

Regardless of the overwhelming effectiveness of preemptive ketamine in animal experiments ^22– 24^, clinical reports are mixed ^25– 29^; some authors have described positive effects ^30^ while others have not ^31^.

While early reviews of clinical findings were mostly contradictory ^26, 32^, there is still conviction among researchers and clinicians in the effectiveness of preemptive analgesia ^5^.

To our knowledge, no prior controlled study has determined the effectiveness of preoperative low-dose iv ketamine as contrasted with placebo in adults after open colon surgery. Thus, this clinical trial was designed to examine the postoperative analgesic effectiveness and opioid-sparing effect of single low-dose iv ketamine in contrast with placebo administered preoperatively.

## Methods

After receiving authorization from the Institutional Ethics Committee (Protocol code MK334037) and according to Helsinki, Tokyo, and Venezia statements, 48 patients undergoing general anesthesia for open colon surgery at the C. Hospitalario Arquitecto Marcide - Profesor Novoa Santos, were studied. This was a randomized controlled clinical trial, ClinicalTrials.gov identifier: NCT02241278.

Study candidates were identified from the surgery schedule and contacted for consent 1–7 days before surgery. All patients gave written, informed consent, after explanation about the objectives, methods and potential risks of the study. Procedures included open colon resections, right hemicolectomy and left hemicolectomy.

Inclusion criteria were age between 18 and 75 years, normal Body Mass Index (18.5–24.9), ASA class I, II or III, elective surgery, surgery time between 60–150 min, understanding of the Visual Analog Scale (VAS), lack of allergies or intolerance to anesthetics and absence of psychiatric illness. Exclusion criteria included cognitive deterioration, inability to use the Patient-Controlled-Analgesia (PCA) device, history of chronic pain syndromes or chronic use of analgesics, sedatives, opioids or steroids, liver or hematologic disease, history of drug or alcohol abuse and intolerance to ketamine or Paracetamol.

Patients were instructed preoperatively on the use of the VAS for pain assessment and the PCA device. The VAS represents a scale with the lowest value as 0 (no pain) and the highest value as 10 (worst imaginable pain).

Randomization was based on computer-produced random-block codes maintained in successively numbered envelopes and organized in a double-blinded manner. Pharmacy-prepared 50 mL solutions containing either ketamine (0.5 mg/kg) or placebo were given to anesthesiologists. The anesthesiologists and patients were not aware of the treatment groups. The investigator, unaware of the treatment groups and not implicated in patient’s intraoperative care, performed postoperative assessments.

All subjects were premedicated with metoclopramide 10 mg and ranitidine 300 mg v.o. the night before and at 07.00 h on the day of surgery, and with diazepam 5–10 mg v.o. the night before surgery. In the operating room, the anesthesiologist administered 0.5 mg/kg of ketamine chlorhydrate in 0.9% saline iv to patients in the ketamine group and 50 mL of 0.9% saline to the control group 30 minutes before surgical incision. Besides routine monitoring, the patients were monitored with spectral entropy through an Entropy Module (M-Entropy TM; Datex-Ohmeda, Helsinki, Finland) and muscle relaxation (M-NMT module).

After premedication with atropine 0.01 mg/kg if necessary, general anesthesia was induced with propofol 1–2 mg/kg (or thiopental 6 mg/kg), remifentanyl at 0.5 µg∙kg ^-1^∙min ^-1^ iv (0.25 µg∙kg ^-1^∙min ^-1^ in patients over 65 years old), muscle relaxation with succinilcoline 1 mg/kg or cisatracurium 0.15 mg/kg. Anesthesia was maintained with nitrous oxide 50% and sevoflurane 0.5–1% in 50% oxygen, remifentanyl in continuous infusion at 0.5–1 µg∙kg ^-1^∙min ^-1^, and neuromuscular blockage with cisatracurium in bolus of 0.06 mg/kg on demand. Anesthesia was adjusted to keep arterial blood pressure and heart rate within 20% of preinduction levels. 30 min before surgical closure, 0.10 mg/kg of morphine was administered iv; a continuous infusion of morphine (PCA) was initiated at 0.015 mg∙kg ^-1^∙h ^-1^ and planned to deliver a bolus of 0.02 mg/kg of morphine on demand, with a lockout interval of 10 min. The infusion of remifentanyl was stopped at the end of surgery. Decurarization if necessary was achieved with atropine 0.01 mg/kg and neostigmine 0.03 mg/kg. The use of opioid reversal agents, different analgesics to the ones studied and other treatments that could interfere with the pain evaluation was not permitted. Patients were extubated in the operating room and moved to the Post-Anesthesia Care Unit (PACU).

Pain severity was evaluated at time 0 (at entrance in the PACU), and at 1, 2, 4, 8, 12, 16, and 24 hours postoperatively. Pain was graded using the VAS. If VAS >5, a rescue dose of Paracetamol 1 gr iv was given as rescue analgesia. The cumulative amounts of morphine administered through the PCA as a basal infusion and the incremental supplemental bolus required by the patient were documented at these same time points. Hemodynamic parameters such as Blood Pressure (BP) systolic, BP diastolic, heart rate and respiratory rate were measured at these same time points. The time interval for the first demand of analgesia and the number of times a rescue dose was injected in the first 24 hours were recorded. Global patient satisfaction (0–3), regarding pain control, was measured 24 hours after the operation. All adverse effects and their characteristics were recorded.

Prior to the study, we calculated the sample size needed for justifying the assumption that postoperative pain (VAS) would be less in the ketamine group than in the control (primary outcome measure). A mean difference in VAS scores of 2.05 (assuming a target of 20.5% reduction in VAS scores) between groups in the first 24 hours postoperatively was defined as clinically relevant. This criterion was based on the results of a previous pilot study at our institution using the same surgical population and the same outcomes. The required sample size to reveal clinically relevant reductions was estimated to be 24 patients per category, giving a statistical power of 0.80 and a type I error protection of 0.05.

We performed a descriptive analysis, presenting the numerical variables as mean ± standard deviation and the categorical variables as integer values and percentages.

Categorical variables were contrasted between groups with the Chi-square test. Numerical variables were compared between groups, after checking the assumption of normal distribution with the Kolmogorov-Smirnov test, with the Student’s t-test test or the Mann-Whitney U-test accordingly.

Variables in the different time points were compared with the Friedman test for related groups. The level of significance was established at P<0.05. Data were examined utilizing SPSS statistical software (v.19.0).

## Results

Data on the effect of preoperative low-dose ketamine in opioid-naïve patients undergoing colon surgery when nitrous oxide is used The file shows in the ketamine and control groups the original patient’s demographic data, intraoperative analgesic data and duration of surgery, the Visual Analog Scale (VAS) scores from time point 0 to time point 7, and the hemodynamic parameters (blood pressures systolic (tad) and diastolic (tas) in mmHg, respiratory rate (fr), and heart rate (fc)) from time point 0 to time point 7.
Variables: Sex: 1=male, 2=female; VAS score between 0 to 10; Remifent (ml): 1 ml=0.01 mg of remifentanil; Morf (mg): morphine administered at the end of surgery.

A total of 48 patients were recruited during 8 months and completed the study. All patients were discharged and no patients presented any severe postoperative complications.

No significant differences were observed between the two groups in demographics such as ASA group (P=1.000), sex (P=0.745) or age (P=0.177). However, they were different in weight (P=0.015) [ Table 1]. The two groups did not deviate in terms of duration of the surgical procedure (P=0.701), intraoperative doses of remifentanyl (P=0.861) or intraoperative doses of morphine (P=0.572). [ Table 2].

**Table 1.** Demographic data.

Variable	Ketamine Group ^a^	Control Group ^a^	*P* value
Age (years)	66.33 ± 11.066	64.38 ± 9.326	0.177
Weight (kg)	69.33 ± 8.676	77.33 ± 12.812	0.015
Gender (male/female)	18/6 75.0%/25.0%	17/7 70.8%/29.2%	0.745
ASA physical status	Median= II	Median= II	1.000
I	0	0	
II	16	16	
III	8	8	

Values are mean ± SD except gender distribution (frequency) and ASA physical status (median value).

^a^ *n* = 24

**Table 2.** Intraoperative analgesic data and duration of surgery.

Variable	Ketamine Group ^a^	Control Group ^a^	*P* value
Duration of surgery (min)	117.71 ± 44.04	122.08 ± 43.73	0.701
Remifentanyl total dose (mg)	1.956 ± 1.094	2.057 ± 1.043	0.861
Morphine total dose (mg)	12.08 ± 2.956	12.13 ± 2.891	0.572

Values are mean ± SD.

^a^ *n* = 24

**Figure 1.**
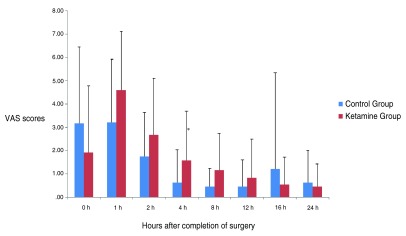
Visual Analog Scale (VAS) pain scores in the groups during the 24 hours after surgery. (Mean ± SD). There were no statistically significant differences between the groups, except at 4 hours of arrival at the PACU (P=0.040)*. We could see a significant effect of time in pain scores for each group separately (P<0.001).

There were no statistically significant differences in VAS scores between the groups, except at 4 hours of arrival to the PACU, when the scores in the ketamine group were higher than in the control group (P=0.040). We could see a significant effect of time in pain scores for each group separately (P<0.001) [ Figure 1]. On arrival at the PACU, pain intensity was higher in the control group, becoming maximal at 1 hour but with higher scores in the ketamine group at this time. We could observe a progressive decrease in pain scores afterwards.

**Figure 2.**
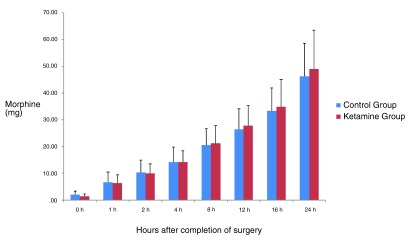
Cumulative patient-controlled analgesia (PCA) morphine consumption in the groups during the 24 hours after surgery. (Mean ± SD). There were no significant differences between groups at any time point (P>0.05). The effect of time on total morphine consumption in the postoperative period was not statistically significant (P>0.05).

No significant differences were assessed between the two groups in cumulative consumption of morphine at any time point during the first postoperative 24 hours (P>0.05 at all time points).The effect of time on morphine consumption through PCA in the entire postoperative period was not statistically significant (P>0.05). ( Figure 2).

**Figure 3.**
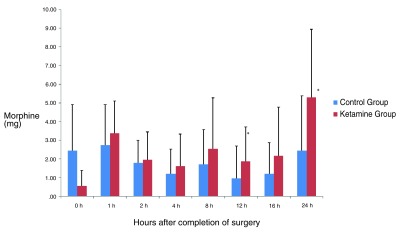
Incremental patient-controlled analgesia (PCA) morphine consumption in bolus in both groups during the 24 hours after surgery. (Mean ± SD). There were no statistically significant differences among groups at any time point, except at 12 h (P=0.013) and 24 h (P=0.002).

The amount of incremental postoperative doses of morphine consumption in bolus from the PCA was comparable in the two groups. We found no statistically significant differences among groups, except at 12 h (P=0.013) and 24 h (P=0.002). It seems the need of additional boluses of morphine over the basal infusion rate of the PCA was slightly higher in the ketamine group at all time points, except immediately after arrival at the PACU ( Figure 3). The total amount of bolus supplements of morphine needed throughout the 24 h was higher in the ketamine group than in the control group (P=0.02). The time to first solicited rescue analgesia was 70 ± 15.491 min in the ketamine group (6 patients) and 44 ± 19.494 min in the control group (5 patients) (P=0.052).

No discordances in patient satisfaction were detected between the groups (P>0.05). The majority of patients rated their pain control as excellent across the 24 h after the operation.

Secondary effects encountered in the ketamine group were nausea (5 patients), urinary retention (1 patient), vomiting (1 patient), incoercible vomiting (1 patient). In the control group they were nausea (3 patients) and urinary retention (2 patients). The differences among groups were not significant (P>0.05). No patient encountered any side effects interpreted as severe ( Table 3).

**Table 3.** Adverse effects.

Variable	Ketamine Group ^a^	Control Group ^a^
Nausea	5	3
Vomiting	1	0
Urinary retention	1	2
Pruritus	0	0
Skin rash	0	0
Respiratory depression	0	0
Incoercible vomiting	1	0

Adverse effects are expressed as number of patients.

^a^ *n* = 24

When evaluating the hemodynamic parameters as an indirect measure of pain, we found the following results. The BP systolic at all time points during the postoperative 24 h was very similar between both groups (P>0.05 at all time points). We could appreciate a slight increase of BP systolic on arrival at the PACU, with a progressive decrease over the 24 h until final stabilization. The BP diastolic was comparable between both groups, with no major statistical deviations, except at 0 h (P=0.026), 8 h (P=0.02) and 24 h (P=0.02), being higher in the ketamine group. These differences did not appear to be clinically significant. The respiratory rate showed no differences between both groups, except at 0 h, being higher in the placebo group (P=0.027), but this difference was not clinically significant. There were no significant differences among groups in heart rate (P>0.05 at all time points).

## Discussion

Demonstration for a clinically significant preemptive analgesic effect of low-dose ketamine is questionable ^33^. Studies have shown a preemptive effect ^15, 21, 28, 34^ and others have not ^18, 26, 27, 31, 35, 36^. Some authors found a 40% decrease in PCA morphine consumption ^21^ and a decrement in hyperalgesia 48 hours ^37^ and 7 days ^38^ after surgery. Barbieri *et al.* ^39^ recorded lower VAS results until 24 hours after elective laparoscopy for ovarian cysts in patients given 1 mg/kg im ketamine before surgery. Fu *et al.* ^15^ contrasted the analgesic effect of a presurgical loading dose (0.5 mg/kg), followed by a constant infusion (10 µg∙kg ^-1^∙min ^-1^) with a single postsurgical dose (0.5 mg/kg). They found a significant decrease in PCA morphine consumption 48 h after surgery in the preemptive group ^26^.

We can deduce from our results that no significant intergroup distinction was encountered in the pain scores. Neither a morphine-sparing effect nor a lower mean supplemental dose of morphine through the PCA was demonstrated at any point in time in the ketamine group.

Despite these results, we observed good analgesia in the immediate postoperative period; as reflected in the pain scores, which were low in both groups and within the maximum limits of VAS 3–4.5; these scores are usually assumed as adequate. As clinically evaluated, there was no activation of the sympathetic nervous system induced by pain in the postoperative period, evidenced by the lack of significant rises in blood pressure, heart rate or respiratory rate. Also, the incidence of adverse effects was low.

Still, we expected that if ketamine had a preemptive analgesic effect, this would have become apparent in the immediate postoperative stage, with significantly lower consumption of morphine and lower pain scores in that group ^18^. However, we cannot unequivocally conclude that ketamine has no preemptive effect from the above information.

A possible explanation is the anesthetic procedure. As debated by Katz ^40^ and Dahl ^41^, examinations on preemptive substances should attempt to clarify whether these substances have a postoperative analgesic effect when clinically pertinent anesthesia, including perioperative opioids, have also been delivered. In all patients, anesthesia was induced and maintained with remifentanil. This may have hidden the preemptive analgesia of ketamine ^26^.

Animal and human investigations propose that the use of adjuvant drugs as part of general anesthesia can act on the central sensitizing effects of surgical stimuli, making it more complex to discern a preemptive effect ^27, 42, 43^. Since even short phases of C-fiber stimulation from surgical injury can lead to sensitization of the central nervous system ^44^, it seems that the constant intraoperative administration of opioids would be superior to reiterated boluses. In our study, the perioperative administration of opioids (remifentanil and morphine) could have blocked, at the presynaptic opioid receptors at the terminals of the C fibers, the release of afferent transmitters involved in pain transmission. Thus, the administration of an NMDA receptor antagonist may have been redundant ^18^.

Moreover, anesthesia was maintained with nitrous oxide in both groups, which has been shown to diminish nociception-induced spinal sensitization in rats ^26, 42, 45^ and to show a preemptive analgesic effect ^13, 42^. Experimental evidence exists in rats that nitrous oxide does block spinal sensitization ^42^, perhaps by the same mechanism as opioids ^46^. However, Goto *et al.* ^42^ demonstrated that halothane and isoflurane ^47^ moderately antagonize this effect equally. Nevertheless, some studies using oxygen/nitrous oxide have exhibited a preemptive analgesic effect ^13, 48, 49^.

Another potential problem was the small dose of drug administered, which might have caused a deficient afferent antinociceptive blockade in the preemptive group. This small dosage has a brief length of action, and central sensitization may have been generated when the pharmacological action of ketamine ended ^26^.

Sensitization is a persistent phenomenon, conditional to the amplitude and length of the nociceptive stimulus. Our study centered on major surgery, where deep noxious stimuli continues during surgery and may even extend postoperatively. The best method to avoid sensitization may be to intercept any pain from the time of incision until final lesion recovering ^26^. Nonetheless, the psychomimetic effects of ketamine limit the clinical value of large-dose ketamine ^27, 50^.

As suggested by the study of Subramaniam *et al.* ^51^, ketamine acts primarily on opened ionic channels to prevent neuroplasticity ^52^. When the drug is given prior to surgery, the channels are not in an open phase, because no noxious stimulus is present. Therefore, it is conceivable that ketamine, because of its brief length of action, must be given as a continuous infusion to inhibit the intraoperative noxious stimuli and the ‘wind up’ occurrence ^15, 51^.

Reza *et al.* ^53^ described in their work that postoperative morphine need was not diminished when 0.5 mg/kg ketamine was given preemptively. Ngan Kee *et al.* illustrated that the postoperative analgesic demand was diminished when 1.0 mg/kg was given in their study ^54^. In spite of this, in other studies 0.5 mg/kg of ketamine was useful for alleviating postoperative pain after abdominal surgery ^5, 15^, and in others the need for analgesia after cesarean section was diminished with administration of a low dosage of 0.15 mg/kg ^55, 56^. In another article the morphine demand was similar in three categories of cesarean section subjects given 0.25, 0.5, or 1.0 mg/kg of ketamine ^57^; hence, it is plausible that the preemptive analgesic action of ketamine might not be dose conditional ^58^.

The choice of surgical procedure may also help to explain our results. Low intensity noxious stimuli during surgery may not incite sufficient central sensitization to create a clear difference between the study groups ^33^. Laskowski *et al.* ^59^ concluded from their study that the efficacy of ketamine was superior in upper abdominal operations, thoracotomy, or if the VAS score was ≥ 7, in contrast to lower abdominal surgery or if the VAS score was < 4. After colon surgery, pain intensity is moderate and may not create adequate highly noxious stimulus to ascertain any clear differences between groups ^26^.

In conclusion, this study failed to exhibit a preemptive analgesic effect of 0.5 mg/kg iv preoperative ketamine, showing no significant advantage on postoperative pain and analgesic consumption. Thus, further comparative and controlled studies of the effects of higher doses in larger study sizes are required before definitive recommendations can be presented.

## Clinical trial registration statement

Patient enrollment for this clinical trial took place during the years 2001 and 2002. The study was not registered prospectively prior to patient enrollment because at the time the trial began enrollment of subjects (years 2001–2002) it was not mandatory the registration of clinical trials on account of the Spanish regulations. The trial was registered on 09/11/2014.

## Data availability

F1000Research: Dataset 1. Data on the effect of preoperative low-dose ketamine in opioid-naïve patients undergoing colon surgery when nitrous oxide is used, 10.5256/f1000research.5258.d35616 ^60^
