# Supplementary material for: Preoperative low-dose ketamine has no preemptive analgesic effect in opioid-naïve patients undergoing colon surgery when nitrous oxide is used - a randomized study
Source: F1000Res. 2014 Sep 23;3:226. [Version 1] doi: 10.12688/f1000research.5258.1 (PMC4309164; doi:10.12688/f1000research.5258.1)
Supplement: Peer review history

# Peer review: Referee response for version 1

**DOI:** 10.5256/f1000research.5605.r7156

**Published:** 2015-01-23

**Authors:** Lisa V. Doan

**Affiliations:** 1 Department of Anesthesiology, NYU School of Medicine, New York, NY, USA

The authors have investigated the role of preoperative ketamine on analgesia in opioid-naive patients undergoing open colon resection. This was a negative study, showing no significant differences in VAS or opioid consumption over the first 24 hours postoperatively.

Overall it was a well conducted study. Perhaps the authors could comment on why epidural analgesia was not utilized for open colon resections (though it is unclear whether this would have affected the outcome measures). The dose of ketamine given preoperatively is low. It would be interesting in future studies to use an infusion of ketamine and to include opioid tolerant patients as a study population.

I have read this submission. I believe that I have an appropriate level of expertise to confirm that it is of an acceptable scientific standard.

# Peer review: Referee response for version 1

**DOI:** 10.5256/f1000research.5605.r6207

**Published:** 2014-09-29

**Authors:** Arthur Atchabahian, Kirsten Boenigk

**Affiliations:** 1 Department of Anesthesiology, NYU Langone Medical Center, New York, NY, USA

This is a useful paper insofar as it seems to indicate that a short-term administration of ketamine does not improve postoperative pain control. The evidence in the literature is contradictory and more data is needed.

Study Design

Type of surgery and anesthetic technique were homogenous. The exclusion of prior chronic pain syndromes or use of pain medication also helps make the study population more homogeneous, although I would question whether there might be some added benefit of ketamine in patients who are taking chronic opioids.

I wish the authors briefly gave the results of their pilot study, especially the standard deviation that was used to calculate the sample size.

As the authors say in their discussion, 0.5 mg/kg ketamine is a relatively low dose. With a t 1/2 of 10 -15 min and an active metabolite that has 1/3 efficacy and also a relatively short t 1/2 of 2.5 h, it is questionable whether the objective of modulating the NMDA receptor response was achieved, and that is probably why there was no benefit of the ketamine administration. Maybe the authors should consider repeating the study using a ketamine infusion.

Second, the administration of a continuous postoperative morphine infusion of 0.015 mg/kg/h may have created some "background noise" and obscured a difference in VAS score or difference in the amount of self-administered morphine.

Article content

The study is well conducted. Data is complete and well analyzed. The discussion is well conducted and addresses the study rationale, findings, and main limitations.

There are a few typos: remifentanil, succinylcholine, psychotomimetic.

We have read this submission. We believe that we have an appropriate level of expertise to confirm that it is of an acceptable scientific standard.
